# Clinic of Prof. Garretson, Hospital of Oral Surgery, Philadelphia

**Published:** 1888-01

**Authors:** Robert S. Ivy


### CLINIC OF PROFESSOR GARRETSON. HOSPITAL OF ORAL SURGERY, PHILADELPHIA.

Reported by Robert S. Ivy, D. D. S.

The clinical service of the Hospital of Oral Surgery was never so full and instructive as at present, seldom less than three hours at a time being required to get through the variety of cases which are presented. The clinic deals with many of the most intricate and complicated performances in surgery, and as a school for study it is one of the best attended and most popular of the hospital services of Philadelphia. Few surgeons have made more operations as to number or more complicated as to performances than has Professor Garretson, and his record of success remains unbroken. Dr. Garretson shows himself something of a fatalist, inasmuch as he never attempts any of his great operations without having at his right hand his chief of the oral clinic, Dr. M. H. Cryer. The two work together as one person, and in moments of danger are recognized as speaking pages to each other by a single look; this is, presumably, the result of their many years of relation.

Dr. Shumwell, chief of Professor Garretson's General Surgical Staff, is another right hand at the clinics, and is never anywhere but in the proper place at moments of emergency.

The anæsthetic exhibitions at this clinic are necessarily without parallel, by reason of the nature of the operations. These are in charge of Professor Dove, and it is no uncommon thing to see an effect, carried and continued to the point of deadness as to external impressions, produced and continued for one or two hours, while all the time care is required to see that the blood from the operation shall not smother the patient.

Amongst the operations recently performed were the following:

Case I. Removal of a tumor occupying a position beneath the carotid artery, consequently overlaid by the vessels contained in the sheath, the sterno-cleido mastoid muscle, the external jugular vein and the integuments.

Case II. Neuralgia in the region of the distribution of the inferior maxillary nerve. The lower portion of the inferior dental branch had been removed on a previous occasion, but the pain still continuing, it was proposed to cut the main branch of the inferior maxillary nerve at the point of its egress from the calavarium, a performance attended by numerous complications, the part being environed by many important vessels and organs. The procedure consisted, first in making an opening through the tissues overlying the perpendicular part of the inferior maxilla, cutting the attachment of the masseter muscle at its insertion and turning it up over the temporal region, thus exposing the greater portion of the external surface of the ramus of the jaw. In making this incision precaution was taken to avoid cutting the duct of steno. The anterior portion of the ramus was next removed. a circular saw revolved by the surgical engine being the instrument employed. The cut being made downwards from the middle of the sigmoid notch to a point opposite the wisdom tooth, thence across the lower third of the bone, the section removed included the coronoid process. In making this cut, a portion of the internal pterygoid muscle was divided, also the temporal muscle at the point of insertion. An incision through the lower head of the external pterygoid muscle next exposed the zygomatic fossa, in which were found the upper portions of the gustatory and inferior dental nerves at their point of division from the main trunk, also the internal maxillary artery and its branches of this region; a portion of the parotid gland was also exposed. The nerve, having been isolated from its surroundings, was cut at its exit through the foramen ovale. Hemorrhage being controlled, the wound was dressed and the patient removed to the ward.

Case III. Ex-section of inferior dental nerve. This operation, on account of its important successful results, is of the most frequent occurrence in this clinic. Its manner is original with Professor Garretson, and consists in making an incision below the lower border of the inferior maxilla, and dissecting away the tissues overlying the bone on its external surface, as far forward as the anterior or mental foramen, in which the facial artery is generally cut and ligated. The next procedure is to remove the outer wall of the canal by means of bur and engine, and to divide the nerve as far back as is necessary, the time required for the operation being only a few minutes. In the case under notice the patient had suffered severe pain in the region of this nerve, the cause being found in a series of neuromata on the main trunk within the canal. One week sufficed to show the patient to the class, completely cured.

